# *In-silico* design of a potential inhibitor of SARS-CoV-2 S protein

**DOI:** 10.1371/journal.pone.0240004

**Published:** 2020-10-01

**Authors:** Grijesh Jaiswal, Veerendra Kumar

**Affiliations:** Amity Institute of Molecular Medicine and Stem Cell Research (AIMMSCR), Amity University, Noida Uttar Pradesh, India; University of Tennessee, UNITED STATES

## Abstract

The SARS-CoV-2 virus has caused a pandemic and is public health emergency of international concern. As of now, no registered therapies are available for treatment of coronavirus infection. The viral infection depends on the attachment of spike (S) glycoprotein to human cell receptor angiotensin-converting enzyme 2 (ACE2). We have designed a protein inhibitor (ΔABP-D25Y) targeting S protein using computational approach. The inhibitor consists of two α helical peptides homologues to protease domain (PD) of ACE2. Docking studies and molecular dynamic simulation revealed that the inhibitor binds exclusively at the ACE2 binding site of S protein. The computed binding affinity of the inhibitor is higher than the ACE2 and thus will likely out compete ACE2 for binding to S protein. Hence, the proposed inhibitor ΔABP-D25Y could be a potential blocker of S protein and receptor binding domain (RBD) attachment.

## Introduction

In December 2019, a novel coronavirus SARS-CoV-2 (also known as 2019-nCoV) caused an outbreak of pulmonary disease in the city of Wuhan, China, and has since spread globally [1,2]. Its genome is about 82% identical to the SARS coronavirus (SARS-CoV). Specifically, the envelope and nucleocapsid proteins of SARS-CoV and SARS-CoV-2 share 96% and 89.6% sequence identities, respectively. Coronaviruses (CoVs) are largest RNA virus family divided into α, β, δ and γ genera. β-coronaviruses are further divided into 4 lineages A, B, C, and D. Both SARS-CoV and SARS-CoV-2 belong to the β-genus and lineage B ((β-B coronaviruses) [3]. The disease caused by SARS-CoV-2 is called Corona Virus Disease 2019 (COVID-19). The SARS-CoV-2 virions are 50−200 nm in diameter [4]. The RNA genome of SARS-CoV-2 consists of 29,811 nucleotides, encodes 29 proteins and phylogenetic analysis suggests bat origin [4,5].

The virus has four structural proteins, known as S (spike), E (envelope), M (membrane), and N (nucleocapsid) proteins. An envelope-anchored SARS-CoV-2 spike (S) glycoprotein facilitates coronavirus entry into host cells [6,7]. The S proteins (~ 1200 aa) are class-I viral fusion proteins and exist as trimers with two of the receptor binding sites (RBDs) facing “up” and the third RBD facing “down”. The monomeric S protein consists of a large ectodomain, a single-pass transmembrane anchor, and a short intracellular tail at C-terminus [8,9]. A total of 22 N-glycosylation sites are present in S protein of SARS-CoV and SARS-CoV-2 at similar positions. However, SARS-CoV S protein has an extra glycosylation site at N370 [10–13]. SARS-CoV-2 spike (S) glycoprotein binds to the cell membrane protein receptor angiotensin-converting enzyme 2 (ACE2) to enter human cells [14,15]. Interestingly, SARS-CoV-2 virus does not use other coronavirus receptors such as aminopeptidase N and dipeptidyl peptidase 4 [1]. Following receptor recognition, the S protein is cleaved into S1 and S2 subunits at furin-like cleavage site [16–18]. The receptor binding domain (RBD) in S1 directly binds to the peptidase domain (PD) of ACE2 [19,20]. RBD consist of a core structure and a receptor-binding motif (RBM), which interacts with the claw-like structure of ACE2 [21,22]. Foremost, the N-terminal α1/α2 helices of ACE2 engage with the RBM motif. The S1 undergoes transient hinge-like motions to become either receptor accessible or inaccessible. RBD binding to cell receptor ACE2 induces the S1 to dissociate from ACE2, prompting the S2 for membrane fusion [18–20].

ACE2 is a type I membrane protein mainly expressed in lungs, heart, kidneys, and intestine [23–25]. Downregulation of ACE2 expression is associated with cardiovascular diseases [26]. The full-length ACE2 consists of an N-terminal PD domain and a collectrin-like domain (CLD) [24]. The CLD domain is followed by a single transmembrane helix and ~40 aa long intracellular segment [24,25]. The primary physiological function of ACE2 is maturation of angiotensin (Ang). The catalytic PD domain cleaves Ang I to produce Ang-(1–9), which is converted to Ang-(1–7) by other enzymes. ACE2 also converts Ang II to Ang-(1–7) directly. These are peptide hormone that controls vasoconstriction and blood pressure [23].

A recent study revealed that human HeLa cells expressing ACE2 become susceptible to SARS-CoV-2 infection [1]. Overexpression of ACE2 enhances the disease severity in mice [27]. Injecting SARS-CoV S protein into mice further worsen the lung injury. However, the lung injury was reversed by blocking the renin-angiotensin pathway [28]. Thus, for SARS-CoV pathogenesis, ACE2 is not only the entry receptor of the virus but it also protects from lung damage. Furthermore, isolated SARS-CoV monoclonal antibodies are not able to neutralize SARS-CoV-2 [29]. Thus, despite the high sequence and structural similarity, there are notable differences between the SARS-CoV and SARS-CoV-2 RBDs. The affinity between the viral RBD and host ACE2 during the initial attachment of virus determines the susceptibility of hosts to the SARS-CoV infection [22,30,31]. Surface plasmon resonance (SPR) study showed that SARS-CoV-2 spike (S) protein binds to ACE2 with 10- to 20-fold higher affinity than the other SARS-CoV S proteins [32], a likely reason for SARS-CoV-2 high infectivity. Thus, viral entry into the host cell is a critical step in viral infection and could be exploited for therapeutics developments.

There is a rapid ongoing search for therapeutics against SARS-CoV-2. Computational approaches have been employed to discover the potential drugs against SARS-CoV-2 [3,33–36]. Drugs targeting either the S protein or main protease have been screened. These approaches have led to the discovery of small molecules with high binding affinities to the aforementioned proteins. However, only a few of these molecules bind at the interface of the RBD−ACE2 complex. For example, hesperidin is predicted to bind at the RBD-ACE2 interface [37]. A short α-helical peptide from the protease domain (PD) of ACE2 showed highly specific and stable blocking to SARS-CoV-2 in a MD simulation study. These peptides blockers cover the full interface of RBD-ACE2 [38].

Till date, there are no clinically approved drugs or antibodies specific for SARS-CoV-2. Here, we have identified a double helical inhibitor that binds very tightly at RBD. The PRODIGY server predicted a dissociation constant (K_D_) of 0.6 nM [39]. MD simulation studies suggest that bound inhibitor is very stable, and it covers the whole RBD-ACE2 interface. Potentially, it can block the binding of RBD to ACE2 and hence it can be used as a potential inhibitor.

## Materials and methods

### Structural analysis

All the protein structures were downloaded from Protein Data Bank (PDB) and their codes are mention as they appear in the manuscript [40]_._ The structures were manually visualised in Coot, Pymol and UCSF Chimera [41–43]. Structural comparison was done using Pymol. The structure coordinates consisting of interface residues RBD (473–508) and ACE2 (21–100) extracted from PDB 6M17 [44] were used as input for structural homolog search using DALI server [45]. Multiple sequence alignment was done using Clustal omega server [46]. All the figures were prepared using Pymol [42].

### Molecular docking

Molecular docking is a powerful tool that can be used to dock the binding of a peptide or ligand at the preferred site and orientation on a macromolecule. Docking programs sample the conformations of the peptide/ligand and then rank these conformations using a scoring function. The RBD fragment (336–518) of spike protein of SARS-CoV-2 (PDB 6M17) was used as a receptor molecule. The truncated peptide (ABP) was docked using HADDOCK2.4 program [47]. HADDOCK2.4 is a freely available platform for observing and analysing protein-protein interactions. Docking by HADDOCK program is driven by prediction of likely residues (called ambiguous interaction restraints (AIRs)) found at the interface. These residues may be active (interacting residue) or passive (nearby interacting residue). Binding interface of RBD and ABP was predicted using CPORT [48] and BIPSPI [49] servers. Before the docking protocol, the pdbs were “cleaned” by removing water and non-bonded ions. The docking results were cross verified using Cluspro 2.0 [50], pyDockWEB [51], and ZDOCK Server [52].

### Molecular Dynamics (MD) simulations

The MD simulation was performed for RBD (aa 436–508) and ΔABP-D25Y complex in Gromacs 2020.2 for 100 ns [53]. Simulation inputs were built using CHARMM-GUI web with CHARMM36 force field [54,55]. The energy minimization and equilibration steps comprising gradual reduction of side chain and backbone restraints was carried out for 250 and 500 ps respectively. Time step was 2fs and the trajectory was saved every 10ps. During production run temperature was maintained at 303 K using velocity rescaling. Bond lengths were constrained with the LINCS algorithm. The pressure was controlled by isotropic coupling using Parrinello-Rahman barostat. A Verlet scheme was used for van der Waals and Particle Mesh Ewald electrostatics interactions within 1.2 nm. Van der Waals interactions were switched above 1.0 nm.

## Results and discussion

### Interface of the ACE2-RBD complex

#### Conformation of ACE2s

**Fig 1:**
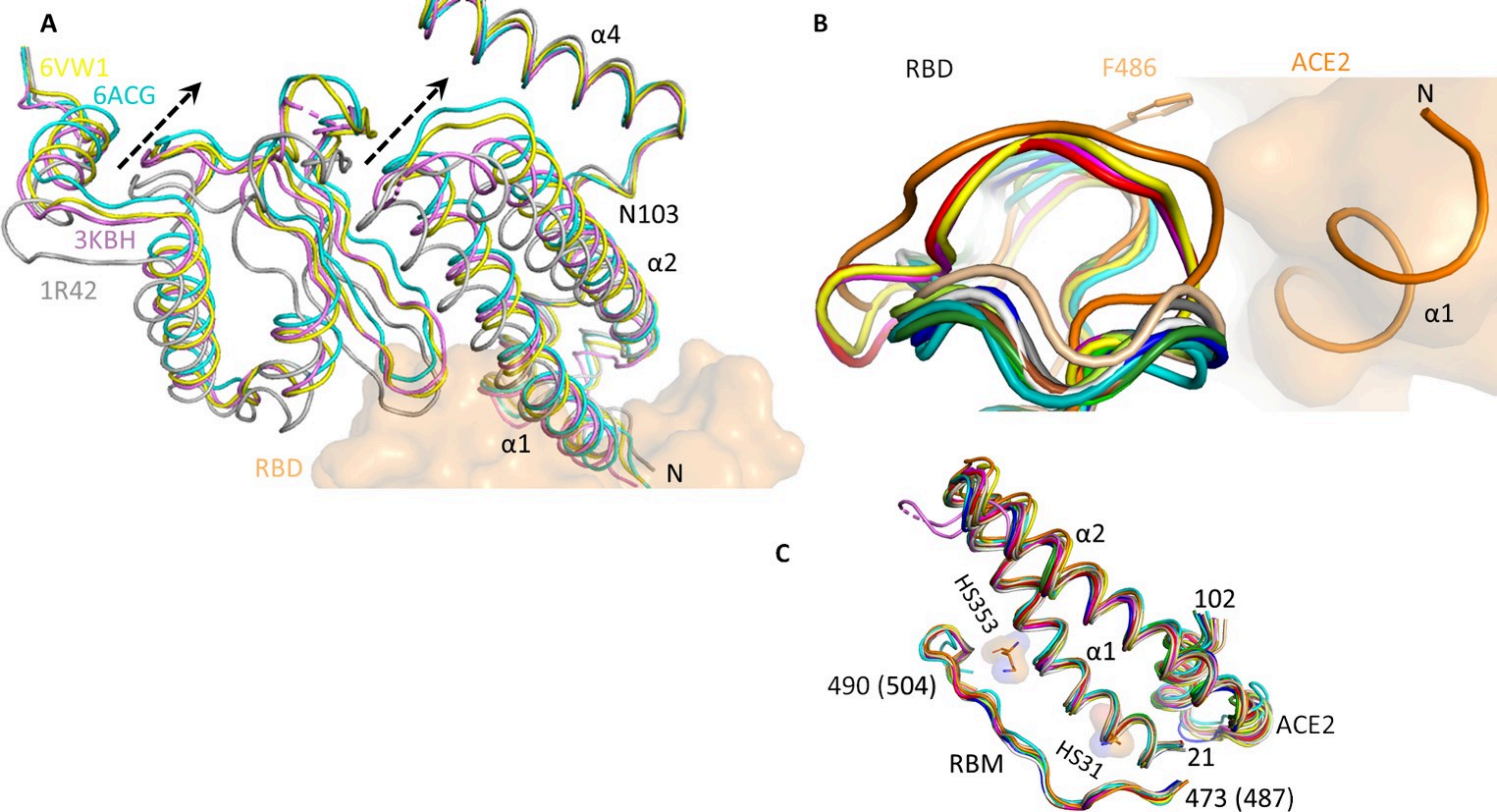
Comparison of coronaviruses RBD and human ACE2. A) In NL63 coronavirus (NL63-CoV) (3kbh, magenta), low pH-treated SARS-CoV (6acg, cyan) and SARS-CoV-2 (6vw1, yellow) RBD bound ACE2 exist in compact conformation compared to unbound ACE2 (1r42, grey). The dotted arrow shows movement of α1 and α2 helices towards α4. B) The capping loop in SARS-CoV-2 (orange) is wider and more flexible compared to other coronaviruses. Because of the wider loop, Phe486 is buried in a hydrophobic pocket at N-terminus of ACE2 α2. C) Binding interfaces of RBD and ACE2 are very similar. SARS-CoV-2 numbering is shown in brackets. PDB used in B) and C) are: 2ajf (grey), 3d0g (forest), 3kbh (violet), 3sci (limon), 3scj (white), 6cs2 (wheat), 6lzg (red), 6m0j (magenta), 6vw1 (yellow), 6m17 (orange), 3scl (teal), 3d0i (brown), 3d0h (green), 3sck (blue) and 6acg (cyan). Two hotspot HS31 and HS353 are shown as surface.

The SARS-CoV-2 genome is about 80% and 96% identical with the genomes of SARS-CoV and bat coronavirus BatCoV RaTG13, respectively [56]. Therefore, the binding pattern between the three viral spikes and human ACE2 is expected to be rather similar. Superimposition of ACE2 from various RBD-ACE2 complexes on unbound ACE2 (pdb 1r42) shows that ACE2 superimposes very well (RMSD 0.4–1.0) (S1A Fig). Interestingly, majority of ACE2s maintain their native state upon binding to RBD, however in NL63 coronavirus (NL63-CoV) (pdb 3kbh), low pH-treated SARS-CoV (pdb 6acg), and SARS-CoV-2 (pdb 6vw1), ACE2 α1/α2 (residues25-102) move “upwards” and come nearer to α4. However, α4 and subsequent structures do not show any conformation shifts (Fig 1A). In these structures, α1/α2 helices rotate about 8.6–11.0° around the pivot Asn103. Further, residues 287–370 also rotate in a similar manner (Fig 1A). Thus, in these structures, ACE2 adopts a more compact conformation compared to unbound ACE2. As cryo-EM structure (6acg) also shows such compact conformation, it is likely not crystal packing artefact but rather reflects a functional state of ACE2. Notably, in case of SARS-CoV-2, only a chimeric RBD consisting of SARS-CoV RBD core and SARS-CoV-2 RBM shows compact conformation [20]. However, it is not clear how the compact conformation of ACE2 will affect the RBD binding.

#### Conformation of RBDs

Comparison of the RBD structures suggest no conformational difference among the RBDs and the binding interfaces (Figs 1C and S1B). Structures of all RBDs are similar apart from the minor differences at the capping loop between 472–488 (SARS-COV-2 numbering). The capping loop in SARS-CoV-2 is wider and more flexible compared to other coronaviruses due to unique amino acid compositions (Fig 1B). The consecutive prolines Pro469 and Pro470 in SARS-CoV loop are replaced by Val483 and Glu484 in SARS-CoV-2. Similarly, Thr468/Gly482, Cys467/Cys480, Lys465/Thr478, Asp463/Gly476, Pro462/Ala475 (SARS-CoV/SARS-CoV-2) mutation makes SARS-CoV-2 loop more flexible. There is an extra amino acid Asn481 in SARS-CoV-2. Thus, the absence of two Pro, presence of Gly and an extra amino acid Asn481 in SARS-CoV-2 capping loop makes it more flexible and “wider”. The wider loop comes close to α1 helix of ACE2 and forms several contacts at N-terminus of the helix, thereby strengthening the RBD-ACE2 complex (Fig 1B). The structure of NL63 coronavirus (NL63-CoV) RBD is unique. NL63-CoV is the only group I coronavirus that uses ACE2 as receptor. It does not share any structural homology with SARS-CoV-2 virus (S1C Fig). NL63-CoV RBD binds only at hotspot 353 with unique interacting residues [57].

#### Conformation of RBD-ACE2 interface

**Fig 2:**
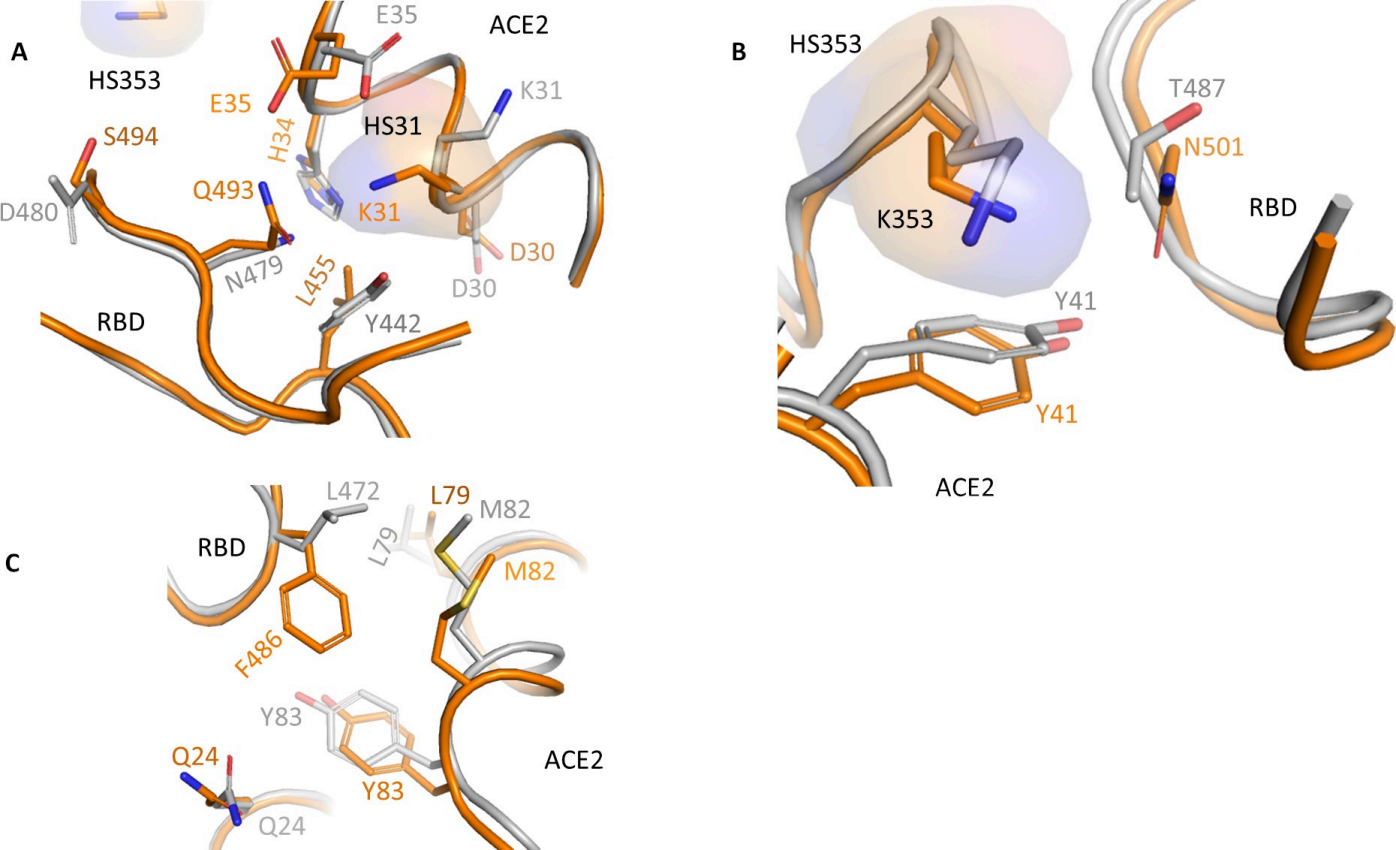
Interaction of SARS-CoV-2 RBD with human ACE2. The unique critical residues A) Leu455, Gln493 and Ser494 of SARS-CoV-2 RBD, B) Asn501 and C) Phe486 enables the SARS-CoV-2 RBD (orange) to interact with human ACE2 stronger compared to SARS-CoV (grey).

The α1-α3 helices (residues 21–102) of ACE2 and the RBM region (residues 473–490 in SARS-CoV and 487–504 in SARS-CoV-2) superimpose very well (Fig 1C). The RMSD and interface RMSD (i-RMSD) of all aligned structures are less than 1.0 Å and 0.5 Å, respectively. While human ACE2 is similar in all structures in Fig 1C, the sequence identity of interface residues of SARS-CoV and SARS-CoV2 is 61%. Thus, ACE2 stabilizes the binding interface on RBD. The 15 residues long β sheet (also called RBM site) surrounded by two capping loops from the S protein interacts with ACE2. The N-terminal α1 helix of the ACE2 (aa 20–52) aligns in parallel with the β sheet of the RBD (Fig 1C). However, the α2 helix of the ACE2 is situated in a different plane and does not interact with RBD. Thus, adaptations of the viral RBD to the N-terminal helix of host ACE2 are critical for viral infections. Mutating one or few residues in RBD could change the binding affinity between the spike protein and ACE2 and thus transform a previously unsusceptible host to a susceptible one [56]. Shang et al, have suggested two virus-binding hot spots in ACE2- hotspot 31 (HS31) and hotspot 353 (HS353) (Figs 1C and 2A). RBM site at these hot spots exhibit more mutations in different corona viruses [20].

Comparison of RBM site of different corona viruses suggest that amino acids Tyr442, Leu472, Asn479, Asp480, and Thr487 (SARS-CoV numbering) affect viral binding to human ACE2. These residues correspond to Leu455, Phe486, Gln493, Ser494, and Asn501 in SARS-CoV-2 RBD, respectively. At hotspot31, Tyr442 interacts with Lys31 of ACE2 in SARS-CoV. In SARS-CoV-2, the corresponding residue Leu455 interacts with Asp30, Lys31, and His34 of ACE2. Because Tyr442 in SARS-CoV is replaced by a less bulky Leu455 in SARS-CoV-2, the salt bridge between Lys31 and Glu35 cannot form (Fig 2A). The residue Gln493 in SARS-CoV-2 RBM forms strong electrostatic interactions with Lys31 and Glu35 in ACE2. Whereas the corresponding residue Asn479 in SARS-CoV interacts only with His34 (Fig 2A). Asn479 is mutated to Lys and Arg in civet and bat, respectively. Its substitution to Lys lowers the affinity by 30-fold [56]. However, its substitution to Gln493 in SARS-CoV-2 enables it to form stronger interaction with ACE2 because of a shorter uncharged polar side chain compared to Lys (Fig 2A).

In contrast, hotspot Lys353 in SARS-CoV-2 is quite similar to SARS-CoV. RBM residue Asn501 (SARS-CoV-2) supports Lys353 in a similar way as Thr487 (SARS-CoV) does. Thr487 in SARS-CoV and Asn501 in SARS-CoV-2 maintain interactions with Tyr41 of ACE2. However, Thr487 substitution to Ser increases the K_D_ by 20-fold [56]. Thus, a bulky polar amino acid is preferred at this position. Its substitution with the bulkier polar amino Asn in SARS-CoV-2 will increase the affinity compared to Thr in SARS-CoV (Fig 2B).

Similarly, mutation Phe486 increases the interaction with Leu79, Met82, and Tyr83 in SARS-CoV-2, whereas corresponding residue Leu472 in SARS-CoV interacts with only Leu79 and Met82 (Fig 2C). As discussed above, Phe486 is in the large flexible loop (480-CNGVEGFNC-488) of RBD. Due to this, Phe486 is buried deep in the hydrophobic pocket formed by residues Gln24, Phe28, Leu79, Met82, Tyr83, and Leu97 (Fig 2C). Lastly, the residue Asp480 in SARS-CoV and the corresponding residue Ser494 in SARS-CoV-2 are not involved in direct interactions with ACE2 (Fig 2A). However, the relatively short and neutral side chain of Ser in SARS-CoV-2 sterically favours the interaction compared to Asp at this position in SARS-CoV.

Thus, the unique amino acids at the specific positions in the SARS-CoV-2 S protein energetically favour the contacts with the ACE2 receptor by stabilizing many interactions. Hence, SARS-CoV-2 S protein shows the highest affinity for human ACE2. Therefore, an inhibitor could be designed to destabilize these interactions to block the association of RBD and ACE2.

### Structural comparison with non-ACE2 homologs

A search for structurally similar interfaces in PDB database was performed using the DALI server [45]. Apart from ACE2, the most structurally similar proteins with known function were *de novo* designed peptides used for drug delivery (Table 1). Interestingly, the designed proteins (pdb 6n9h and 6naf) bind the drug amantadine (a C3 symmetric small molecule) and are called ABP (amantadine-binding protein) [58]. Despite low sequence identity, we observe close alignment of ABP with the α1/α2 helices of ACE2 (S2 Fig). Thus, ABP is structurally very similar to the RBD binding region of ACE2. Hence, ABP might compete with ACE2 for binding with SARS-CoV-2 S protein. The fragment α1/α2 (residues 21–100) from PD domain of ACE2 has been suggested as inhibitor for the RBD protein [38]. MD simulation studies have confirmed a strong binding of ACE2 peptides to RBD of SARS-CoV-2. Thus, using this type of peptides with drugs attached to them could prove to be an efficient treatment strategy against the COVID-19. Such peptides together with bound drug could be attached to the surface of nanoparticles.

**Table 1:** Structural homologs of α1/α2 helices of human ACE2.

PDB	Dali score	RMSD Ca	No of residues aligned	Sequence identity %
AMANTADINE-BINDING PROTEIN 6naf	7.5	1.9	67	6
DESIGN CONSTRUCT 2L6HC3_13 5j0h	7.4	2.1	67	7
AMANTADINE-BINDING PROTEIN 6n9h	7.3	2.0	67	6
PRO-2.5 6msr	7.0	2.5	69	16
PRO-2.3 6msq	6.8	2.3	68	18

### Design of peptide inhibitor

**Fig 3:**
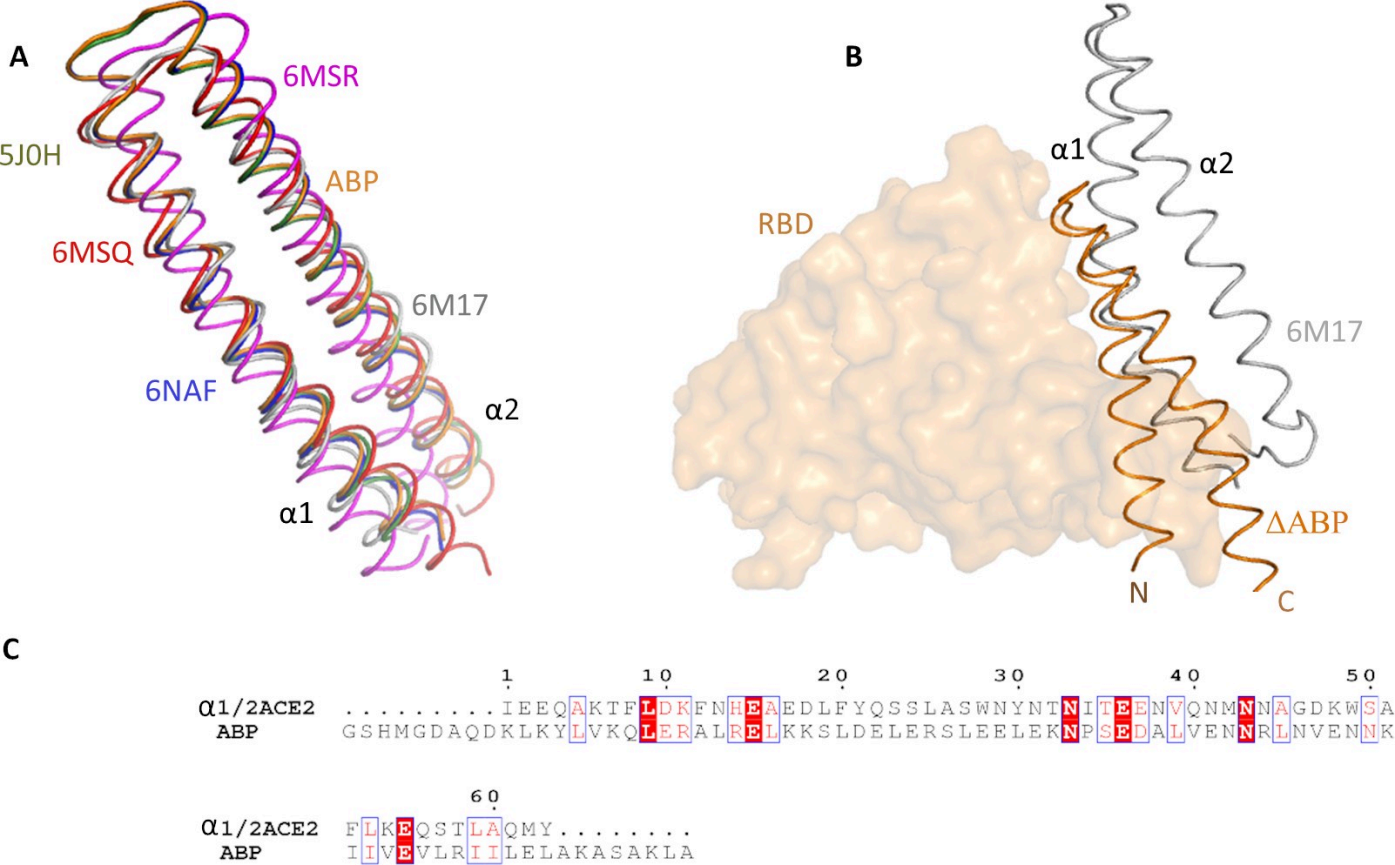
Structural homolog of human ACE2. A) The α1 and α2 helices of ACE2 (6m17, grey) are structurally very similar to the *de novo* designed peptides 5j0h (forest), 6naf (blue), 6msq (red), 6msr (magenta), and 6n9h (or ABP; orange). B) The ΔABP inhibitor binds at the SARS-CoV-2 RBM site. C) Sequence alignment of α1 and α2 with ABP peptide sequence. Similar residues are shown in red, whereas identical sequences are highlighted as white letters on a red background. This diagram was prepared using the program ESPript [67].

**Fig 4:**
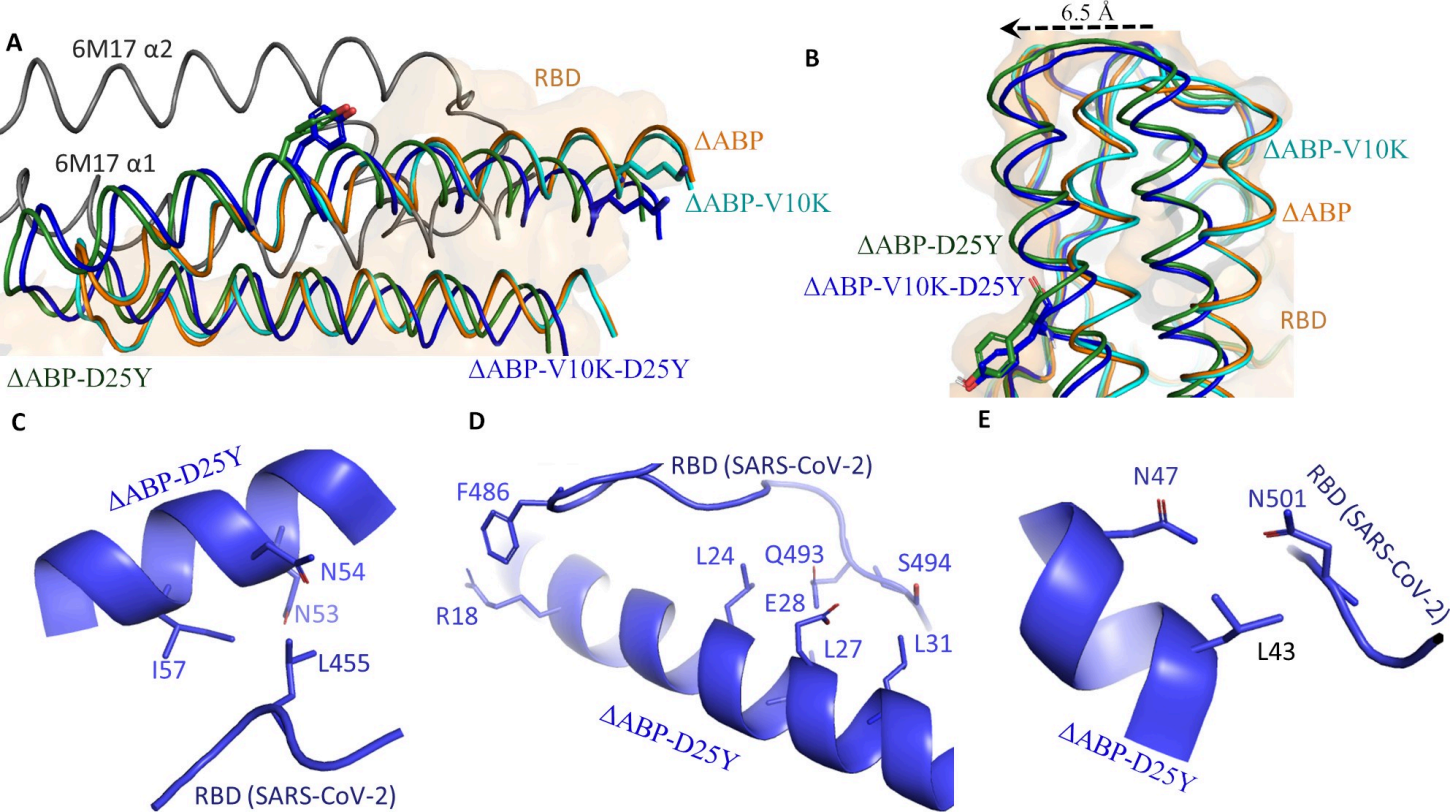
Interaction of ΔABP-D25Y with SARS-CoV-2 RBD. A) HADDOCK results show that all ΔABP peptides bind at the RBM site of the coronavirus S proteins. The α1/α2 helices of ΔABP interact with RBD whereas only α1 of human ACE2 binds with RBD. B) The mutant inhibitors ΔABP-D25Y and ΔABP-V10K-D25Y are shifted laterally. Specific RBD amino acids C) Leu455, D) F486, Gln493 and Ser494 E) Asn501 interactions with ΔABP-D25Y.

Based on the above analysis, we set to design a peptide inhibitor that can interact with the viral RBD and prevents its binding to ACE2. The variants of ABP were tested for their ability to bind the S protein. BIPSPI server [49] predicted the binding interface at the RBM site (Table 3). The peptides were docked on various docking server to confirm the binding. In all the docking studies ABP binds on the RBD surface where α1 helix of ACE2 is known to bind. However, ABP is slightly longer than ACE2 peptides. So, to complement the RBM surface, we removed 4 residues from N-and 10 residues from C-terminus of ABP (called ΔABP). Bound peptide forms strong interaction with RBD and specially interferes with critical residues involved in binding (Figs 3B and 4). PRODIGY server predicted a dissociation constant (K_D_) of 2.8 nM (ΔG -11.7 kcal mol^-1^). A close examination of aligned structure of ΔABP and α1/α2 helices of ACE2 suggests that both have similar side chains except Val10/Lys26 and Asp25/Tyr41 (ΔABP/ACE2) (S2 Fig).

Thus, we sequentially introduce the aforementioned mutations into ΔABP (ΔABP-V10K, ΔABP-D25Y and ΔABP-V10K-D25Y) and docked the mutant peptides into RBD (Table 2). The interaction restraints were generated using CPORT and BIPSPI servers [48,49]. As expected from structural homology of ΔABP with α1/α2 of ACE2, the interaction restraints were same as CoV-SARS-2 RBD and ACE2 complex interface (Table 3). RBM site residues of S protein and all residues of ΔABP were used as active residues for the HADDOCK computation. Best complex was selected based on cluster size, HADDOCK score, and electrostatic energy. HADDOCK score is computed as a weighted sum of van de Waals, electrostatic, desolvation, and ambiguous interaction restraints energies. Electrostatic and van der Waal forces dominate in RBD and ΔABP interactions. The ΔABP-D25Y showed the best HADDOCK score and binding affinity for RBD (Table 2). Interaction of ΔABP-D25Y with SARS-CoV-2 RBD (S1 File) is discussed below.

**Table 2:** HADDOCK docking statics and PRODIGY server binding affinity of various ΔABPs.

Peptides	HADDOCK score*	Van der Waals energy (E_vdw_) (kcal mol^-1^)	Electrostatic energy (E_elec_) (kcal mol^-1^)	Desolvation energy (E_desol_) (kcal mol^-1^)	Restraints violation energy (E_AIR_) (kcal mol^-1^)	PRODIGY dissociation constant nM (G kcal mol^-1^)
**ΔABP**	-70.1 +/- 5.8	-70.4 +/- 4.4	-232.8 +/- 28.4	-16.7 +/- 2.1	636.2 +/- 24.13	2.5 (-11.7)
**ΔABP-V10K**	-82.9 +/- 9.0	-79.2 +/- 8.7	-229.4 +/- 69.0	-15.3 +/- 5.3	574.5 +/- 93.83	2.8 (-11.7)
**ΔABP-D25Y**	-86.1 +/- 8.8	-73.7 +/- 12.9	-231.3 +/- 88.3	-17.8 +/- 6.0	517.2 +/- 74.58	0.6 (-12.6)
**ΔABP- V10K -D25Y**	-78.3 +/- 5.2	-75.5 +/- 12.9	-217.6 +/- 49.9	-17.1 +/- 2.8	578.2 +/- 80.56	0.38 (-12.8)

*HADDOCK score: 1.0 E_vdw_ + 0.2 E_elec_ + 1.0 E_desol_ + 0.1 E_AIR._

**Table 3:** BIPSPI server prediction of binding interface of RBD and ΔABP peptide.

Receptor binding domain (RBD) S glycoprotein SARS-CoV-2			ΔABP			residue	score	expected precision	residue	score	expected precision
505	4.0115	0.8119	62	1.9040	0.6756
493	2.3656	0.6998	61	1.2622	0.6512
489	2.0094	0.6796	48	1.1550	0.6453
486	1.0445	0.6367	6	1.0845	0.6404
487	0.6196	0.6028	8	1.0110	0.6329
485	0.5722	0.5980	47	0.8701	0.6236
504	0.5440	0.5969	43	0.7737	0.6213
506	0.5066	0.5880	44	0.5361	0.5948
481	0.4291	0.5765	51	0.4526	0.5780
490	0.3758	0.5706	55	0.4253	0.5765
488	0.3644	0.5688	59	0.4038	0.5756
494	0.3605	0.5663	65	0.3876	0.5733
502	0.3217	0.5560	58	0.3799	0.5713
492	0.2965	0.5497	63	0.3701	0.5701
503	0.2851	0.5454	9	0.3543	0.5643
483	0.2679	0.5390	64	0.3465	0.5632
484	0.2589	0.5363	7	0.3443	0.5616
455	0.2498	0.5327	54	0.3418	0.5611
480	0.2000	0.5096	38	0.3357	0.5590
447	0.1947	0.5073	28	0.3020	0.5498

### Interaction of ΔABP-D25Y

HADDOCK docking analysis suggests that all ΔABPs bind at the RBM site of S protein. The bound ΔABPs cover the whole surface of the RBM site. However, ΔABPs binding does not exactly overlap with the α1/α2 of ACE2 (Figs 3B and 4A). The position of α1 helix of both ACE2 and ABP is very similar. However, the α2 helix of ACE2 and ΔABP are situated in a different plane and do not overlap with each other (Figs 3B and 4A). The α2 helix of ACE2 does not form direct contact with the virus RBD whereas both helices of ΔABP interact with the RBD. Thus, ΔABP covers a larger surface area on RBD than ACE2. Interestingly, Asp25Tyr mutation in ΔABP-D25Y and ΔABP-V10K-D25Y shifts the peptides laterally by ~6.5 Å. However, the bound peptide remains in same plane as ΔABP and ΔABP-V10K (Fig 4B).

**Fig 5:**
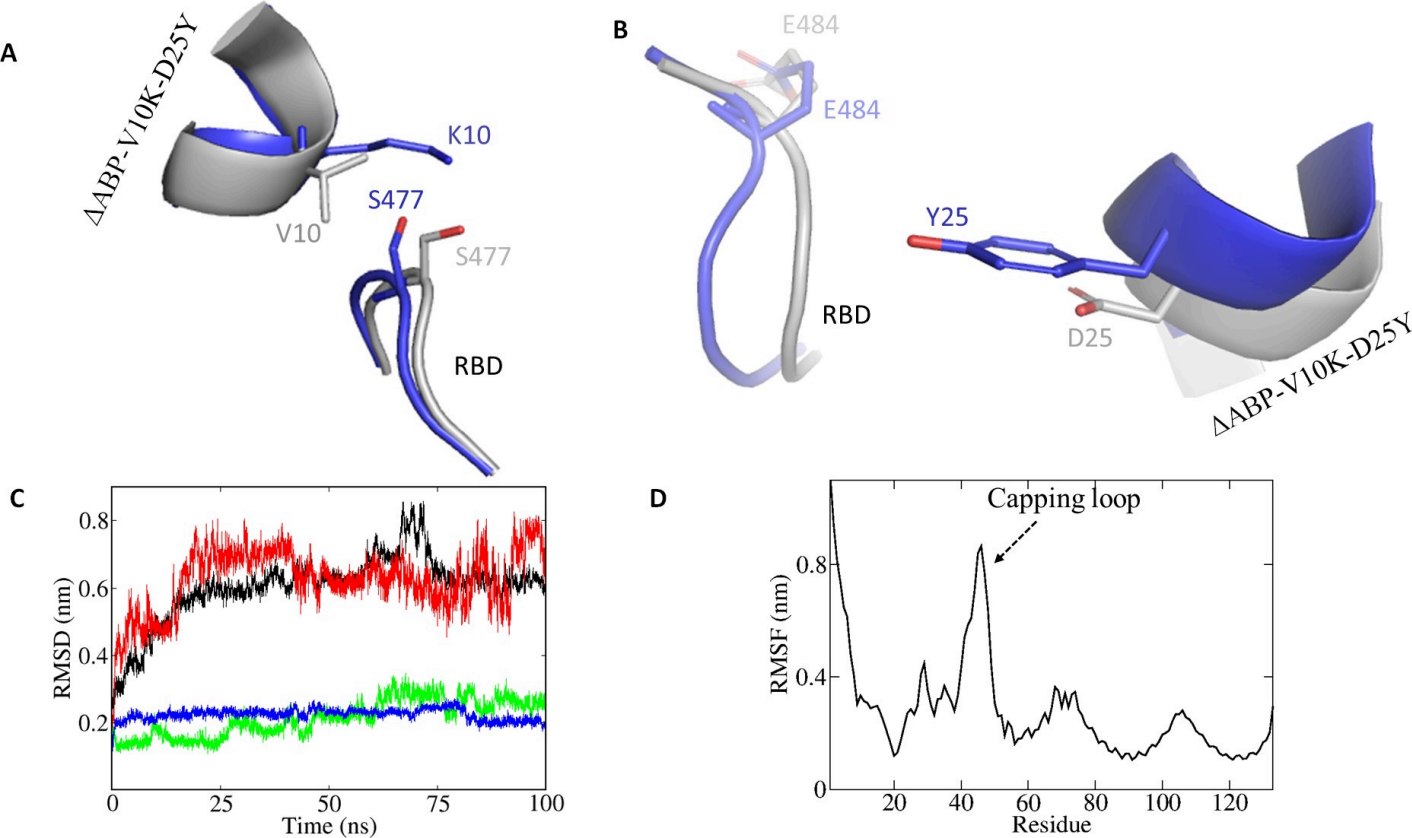
The mutant inhibitor interaction with RBD. A) Val10Lys mutation enables the peptide to interact with Ser477 of RBD. B) Asp25Tyr mutation interacts with Glu484. C) RMSD curves of SARS-CoV-2 RBD and ΔABP-D25Y complex (black), RBD capping loop (residues 472–488; red), RBD interface (residues 489–499; green) and ΔABP-D25Y interface (residues 17–34; blue) during the 100 ns simulation. D) Averaged root-mean-square fluctuation for each amino acid in SARS-CoV-2 RBD (denoted by residue 1–73) and ΔABP-D25Y (denoted by residue 74–133) complex. Capping loop at RBM site is highly flexible.

The Leu455 of SARS-CoV-2 RBD is buried deep in the hydrophobic pocket. The Leu455 forms strong hydrophobic interactions with Asn53, Asn54, and ILE57. (Fig 4C). The N- terminus of the peptide makes direct contact with the RBD loop (residues 470–489). Particularly, Phe486 residue makes cation-π interactions with Arg18 (Fig 4D). Similarly, the Gln493 interacts hydrophobically with Leu24, Leu27, Glu28, and Leu31 of the peptides. The adjacent Ser494 is also in close contact with Leu31 of the peptide (Fig 4D). The residue Asn501 contacts with Leu43 and Asn47 of ΔABP-D25Y (Fig 4E). The mutated residues Asp25Tyr and Val10Lys in ΔABP-V10K-D25Y interact with Glu484 and Ser477, respectively (Fig 5A and 5B). Thus, the individual mutations Val10Lys and Asp25Tyr improve the affinity. However, the combined effect of mutations Val10Lys and Asp25Tyr is detrimental and decreases the affinity of the double mutant inhibitor to SARS-CoV-2 RBD.

Thus, based on the HADDOCK score, the ΔABP-D25Y interacts very strongly with S protein at the RBM site of viral S protein. The biolayer interferometry and surface plasmon resonance experiment showed the dissociation constant (K_D_), 1.2 nM and 4.7 nM respectively, for ACE2 and ectodomain S protein interaction [10,59]. The PRODIGY server predicted K_D_ value of ACE2 binding to RBD is 10 nM (ΔG -10.9 kcal mol^-1^). The slight difference between experimental and calculated K_D_ values may be because full-length S protein was used in experiments. Since full length S protein and ACE2 complex structure is not solved yet, so we could not calculate the K_D_ for the complex. The PRODIGY server predicted the dissociation constant (K_D_) of ΔABP-D25Y to RBD, 0.6 nM at 25.0°C (ΔG -12.6 kcal mol^-1^). Thus, ΔABP-D25Y binds RBD with much higher affinity. Particularly, ΔABP-D25Y directly contacts the unique residues of RBM involved in interaction with ACE2. Protein-protein interactions (PPIs) have emerged as important targets in medicinal chemistry. PPI inhibitors can bind over larger surface area and are therefore more efficient than small chemical ligands [60]. The peptides HR1P and HR2P have been used to inhibit the MERS-CoV infection particularly the replication and fusion steps in calu-3 and HFL cells [61]. Similarly, three peptides SARS_WW-III_, SARS_WW-IV_ and MHV_WW-IV_ showed antiviral activity [62]. Mutant mucropin-M1 peptide (LFRLIKSLIKRLVSAFK) is derived from antimicrobial peptide mucropin ((LFGLIPSLIGGLVSAFK) AMP by replacing several Gly and Pro residues either with Lys or Arg. Mutant mucropin-M1 was active against SARS-CoV inhibiting its replication [63]. The two peptides K12 and K29 obtained from nsp10 of SARS-CoV inhibited the replication of SARS-CoV [64]. Similarly, the peptide OC43-HR2P and its optimized form EK1 are derived from the HR2 domain of human coronavirus (HCoV-OC43). The EK1 showed broad viral membrane and host membrane fusion inhibitory activity against multiple human CoVs. Interestingly, the EK1 forms stable helical complex with HR1 domain of S protein of various coronaviruses [65]. Thus, short α helical peptides have proved effective therapeutics against coronaviruses.

### MD simulation

Root mean square deviation (RMSD) measures the spatial differences between a starting and simulated structure. Similarly, root mean square fluctuation (RMSF) measures the average displacement of a particle over time from a reference position. The RMSF is useful to identify the rigid and flexible regions in protein. The RMSD and RMSF were calculated between initially docked structure and simulated structure for all protein atoms (Fig 5C and 5D). The RBD and ΔABP-D25Y complex has overall low RMSD (~ 0.5 nm). RMSD increases in the beginning up to 18 ns. The plateau after 18 ns indicates that the complex does not fluctuates too much from reference (Fig 5C). A sudden jump in RMSD at around 70 ns is possibly due to the flexibility of capping loop (Fig 5C) and end terminal residues. However, the possibility of insufficient sampling due to rough energy landscapes cannot be ruled out [66]. The RBD capping loop (residues 472–488) is flexible during simulation (Fig 5D). The RMSD of interface of RBD and ΔABP-D25Y complex is very low (~ 0.2 nm). In contrast, the RMSD of capping loop is very close to the overall complex (Fig 5C). So, the overall RMSD (~ 0.8 nm) is mainly contributed by the capping loop and similar flexible region of the complex. However, the interface of the complex is stable, and the inhibitor does not dissociate from RBD. It is important for inhibitors to have complementary conformation matching its target to have better affinity. An inhibitor should have selective binding and low RMSD for the critical amino acids. Such peptides can be easily be chemically synthesized or, alternatively, can be produced in large quantity by recombinant overexpression. Affinity can be further improved by attaching many such inhibitors on the surface of nanoparticles, dendrimers, and clusters. Though we have verified our findings by various in silico methods, further experimental verification is warranted.

## Conclusion

By using computational methods, a protein inhibitor was designed to target the viral S protein. The inhibitor was structurally based on the α helical region of ACE2 at the S protein binding interface. Docking and MD simulation studies suggest that the ΔABP-D25Y could acts as a potential blocker of SARS-CoV-2 infection. It covers the entire RBM surface and its shape is complementary to the RBD groove. Thus, it fits nicely at the RBD groove and block S proteins attachment to ACE2. Predicted binding affinity of the inhibitor is higher than the ACE2 and thus will compete with ACE2 for binding with S protein. Thus, proposed inhibitor could be a potential blocker of S protein and RBD association and thereby hinder virus entry to cells.

## Supporting information

S1 Fig Superimposition of ACE2 from various RBD-ACE2 complexes on unbound ACE2 (grey, 1r42). RBD is shown as orange surface. B) alignment of various RBD on pdb 2ajf RBD (grey). Human ACE2 is shown as orange surface. C) NL63 coronavirus (NL63-CoV) RBD structure is unique among coronaviruses. NL63-CoV RBD (3hbk, magenta) binds at hotspot 353 with unique interacting residues. PDB used in A) and B) are: 3d0g (forest), 3kbh (violet), 3sci (limon), 3scj (white), 6cs2 (wheat), 6lzg (red), 6m0j (magenta), 6vw1 (yellow), 6m17 (orange), 3scl (teal), 3d0i (brown), 3d0h (green), 3sck (blue) and 6acg (cyan).
(TIF)

S2 Fig A close examination of the aligned structure of ΔABP (orange) and α1/α2 helices of ACE2 (grey). Inset1: Close up view of residue Val10/Lys26. Inset 2: Close up view of Asp25/Tyr41.
(TIF)

S1 File (PDB)

## References

[1] Zhou P, Yang X lou, Wang XG, Hu B, Zhang L, Zhang W, et al A pneumonia outbreak associated with a new coronavirus of probable bat origin. Nature. 2020 10.1038/s41586-020-2012-7 32015507 PMC7095418

[2] Wu F, Zhao S, Yu B, Chen YM, Wang W, Song ZG, et al A new coronavirus associated with human respiratory disease in China. Nature. 2020;579: 265–269. 10.1038/s41586-020-2008-3 32015508 PMC7094943

[3] Zhou Y, Hou Y, Shen J, Huang Y, Martin W, Cheng F. Network-based drug repurposing for novel coronavirus 2019-nCoV/SARS-CoV-2. Cell Discovery. 2020 10.1038/s41421-020-0153-3 32194980 PMC7073332

[4] Zhu N, Zhang D, Wang W, Li X, Yang B, Song J, et al A novel coronavirus from patients with pneumonia in China, 2019. New England Journal of Medicine. 2020;382: 727–733. 10.1056/NEJMoa2001017 31978945 PMC7092803

[5] Ren LL, Wang YM, Wu ZQ, Xiang ZC, Guo L, Xu T, et al Identification of a novel coronavirus causing severe pneumonia in human: a descriptive study. Chinese medical journal. 2020 10.1097/CM9.0000000000000722 32004165 PMC7147275

[6] Li F. Structure, Function, and Evolution of Coronavirus Spike Proteins. Annual Review of Virology. 2016 10.1146/annurev-virology-110615-042301 27578435 PMC5457962

[7] Wan Y, Shang J, Graham R, Baric RS, Li F. Receptor Recognition by the Novel Coronavirus from Wuhan: an Analysis Based on Decade-Long Structural Studies of SARS Coronavirus. Journal of Virology. 2020 10.1128/jvi.00127-20 31996437 PMC7081895

[8] Bosch BJ, van der Zee R, de Haan CAM, Rottier PJM. The Coronavirus Spike Protein Is a Class I Virus Fusion Protein: Structural and Functional Characterization of the Fusion Core Complex. Journal of Virology. 2003 10.1128/jvi.77.16.8801-8811.2003 PMC167208 12885899

[9] Gao J, Lu G, Qi J, Li Y, Wu Y, Deng Y, et al Structure of the Fusion Core and Inhibition of Fusion by a Heptad Repeat Peptide Derived from the S Protein of Middle East Respiratory Syndrome Coronavirus. Journal of Virology. 2013 10.1128/jvi.02433-13 24067982 PMC3838252

[10] Walls AC, Park YJ, Tortorici MA, Wall A, McGuire AT, Veesler D. Structure, Function, and Antigenicity of the SARS-CoV-2 Spike Glycoprotein. Cell. 2020 10.1016/j.cell.2020.02.058 32155444 PMC7102599

[11] Simmons G, Reeves JD, Rennekamp AJ, Amberg SM, Piefer AJ, Bates P. Characterization of severe acute respiratory syndrome-associated coronavirus (SARS-CoV) spike glycoprotein-mediated viral entry. Proceedings of the National Academy of Sciences of the United States of America. 2004 10.1073/pnas.0306446101 15010527 PMC384725

[12] Kumar S, Maurya VK, Prasad AK, Bhatt MLB, Saxena SK. Structural, glycosylation and antigenic variation between 2019 novel coronavirus (2019-nCoV) and SARS coronavirus (SARS-CoV). VirusDisease. 2020 10.1007/s13337-020-00571-5 32206694 PMC7085496

[13] Walls AC, Tortorici MA, Frenz B, Snijder J, Li W, Rey FA, et al Glycan shield and epitope masking of a coronavirus spike protein observed by cryo-electron microscopy. Nature Structural and Molecular Biology. 2016 10.1038/nsmb.3293 27617430 PMC5515730

[14] Li W, Moore MJ, Vasllieva N, Sui J, Wong SK, Berne MA, et al Angiotensin-converting enzyme 2 is a functional receptor for the SARS coronavirus. Nature. 2003 10.1038/nature02145 14647384 PMC7095016

[15] Tai W, He L, Zhang X, Pu J, Voronin D, Jiang S, et al Characterization of the receptor-binding domain (RBD) of 2019 novel coronavirus: implication for development of RBD protein as a viral attachment inhibitor and vaccine. Cellular and Molecular Immunology. 2020 10.1038/s41423-020-0400-4 32203189 PMC7091888

[16] Hoffmann M, Kleine-Weber H, Pöhlmann S. A Multibasic Cleavage Site in the Spike Protein of SARS-CoV-2 Is Essential for Infection of Human Lung Cells. Molecular Cell. 2020 10.1016/j.molcel.2020.04.022 32362314 PMC7194065

[17] Xia S, Zhu Y, Liu M, Lan Q, Xu W, Wu Y, et al Fusion mechanism of 2019-nCoV and fusion inhibitors targeting HR1 domain in spike protein. Cellular and Molecular Immunology. 2020 10.1038/s41423-020-0374-2 32047258 PMC7075278

[18] Coutard B, Valle C, de Lamballerie X, Canard B, Seidah NG, Decroly E. The spike glycoprotein of the new coronavirus 2019-nCoV contains a furin-like cleavage site absent in CoV of the same clade. Antiviral Research. 2020 10.1016/j.antiviral.2020.104742 32057769 PMC7114094

[19] Yan R, Zhang Y, Li Y, Xia L, Guo Y, Zhou Q. Structural basis for the recognition of SARS-CoV-2 by full-length human ACE2. Science. 2020 10.1126/science.abb2762 32132184 PMC7164635

[20] Shang J, Ye G, Shi K, Wan Y, Luo C, Aihara H, et al Structural basis of receptor recognition by SARS-CoV-2. Nature. 2020 10.1038/s41586-020-2179-y 32225175 PMC7328981

[21] Li F, Li W, Farzan M, Harrison SC. Structural biology: Structure of SARS coronavirus spike receptor-binding domain complexed with receptor. Science. 2005 10.1126/science.1116480 16166518

[22] Li W, Greenough TC, Moore MJ, Vasilieva N, Somasundaran M, Sullivan JL, et al Efficient Replication of Severe Acute Respiratory Syndrome Coronavirus in Mouse Cells Is Limited by Murine Angiotensin-Converting Enzyme 2. Journal of Virology. 2004 10.1128/jvi.78.20.11429-11433.2004 PMC521845 15452268

[23] Ferrarlo CM. ACE2: More of Ang-(1–7) or less Ang II? Current Opinion in Nephrology and Hypertension. 2011 10.1097/MNH.0b013e3283406f57 21045683 PMC5826562

[24] Towler P, Staker B, Prasad SG, Menon S, Tang J, Parsons T, et al ACE2 X-Ray Structures Reveal a Large Hinge-bending Motion Important for Inhibitor Binding and Catalysis. Journal of Biological Chemistry. 2004 10.1074/jbc.M311191200 14754895 PMC7980034

[25] Donoghue M, Hsieh F, Baronas E, Godbout K, Gosselin M, Stagliano N, et al A novel angiotensin-converting enzyme-related carboxypeptidase (ACE2) converts angiotensin I to angiotensin 1–9. Circulation research. 2000 10.1161/01.res.87.5.e1 10969042

[26] Imai Y, Kuba K, Penninger JM. The discovery of angiotensin-converting enzyme 2 and its role in acute lung injury in mice. Experimental Physiology. 2008 10.1113/expphysiol.2007.040048 18448662 PMC7197898

[27] Yang XH, Deng W, Tong Z, Liu YX, Zhang LF, Zhu H, et al Mice transgenic for human angiotensin-converting enzyme 2 provide a model for SARS coronavirus infection. Comparative Medicine. 2007. 17974127

[28] Imai Y, Kuba K, Rao S, Huan Y, Guo F, Guan B, et al Angiotensin-converting enzyme 2 protects from severe acute lung failure. Nature. 2005 10.1038/nature03712 16001071 PMC7094998

[29] Tian X, Li C, Huang A, Xia S, Lu S, Shi Z, et al Potent binding of 2019 novel coronavirus spike protein by a SARS coronavirus-specific human monoclonal antibody. Emerging Microbes and Infections. 2020 10.1080/22221751.2020.1729069 32065055 PMC7048180

[30] Li F. Receptor Recognition Mechanisms of Coronaviruses: a Decade of Structural Studies. Journal of Virology. 2015 10.1128/jvi.02615-14 25428871 PMC4338876

[31] Belouzard S, Millet JK, Licitra BN, Whittaker GR. Mechanisms of coronavirus cell entry mediated by the viral spike protein. Viruses. 2012 10.3390/v4061011 22816037 PMC3397359

[32] Wrapp D, Wang N, Corbett KS, Goldsmith JA, Hsieh CL, Abiona O, et al Cryo-EM structure of the 2019-nCoV spike in the prefusion conformation. Science. 2020 10.1126/science.aax0902 32075877 PMC7164637

[33] Wu C, Liu Y, Yang Y, Zhang P, Zhong W, Wang Y, et al Analysis of therapeutic targets for SARS-CoV-2 and discovery of potential drugs by computational methods. Acta Pharmaceutica Sinica B. 2020 10.1016/j.apsb.2020.02.008 32292689 PMC7102550

[34] Mirza, M. U.; Froeyen M. Structural Elucidation of SARS-CoV-2 Vital Proteins: Computational Methods Reveal Potential Drug Candidates Against Main Protease, Nsp12 RNA-dependent RNA Polymerase and Nsp13 Helicase. Preprints. 2020. 10.20944/preprints202003.0085.v1 PMC7187848 32346490

[35] Khan RJ, Jha RK, Amera GM, Jain M, Singh E, Pathak A, et al Targeting SARS-CoV-2: A Systematic Drug Repurposing Approach to Identify Promising Inhibitors Against 3C-like Proteinase and 2’-O-RiboseMethyltransferase. Journal of biomolecular structure & dynamics. 2020 10.1080/07391102.2020.1753577 32266873 PMC7189412

[36] Chandel V, Raj S, Rathi B, Kumar D. In silico identification of potent FDA approved drugs against Coronavirus COVID-19 main protease: A drug repurposing approach. Chemical Biology Letters. 2020.

[37] Meneguzzo F, Ciriminna R, Zabini F, Pagliaro M. Review of Evidence Available on Hesperidin-Rich Products as Potential Tools against COVID-19 and Hydrodynamic Cavitation-Based Extraction as a Method of Increasing Their Production. Processes. 2020;8: 549 10.3390/PR8050549

[38] Han Y, Král P. Computational Design of ACE2-Based Peptide Inhibitors of SARS-CoV-2. ACS Nano. 2020 10.1021/acsnano.0c02857 32286790 PMC7163933

[39] Xue LC, Rodrigues JP, Kastritis PL, Bonvin AM, Vangone A. PRODIGY: A web server for predicting the binding affinity of protein-protein complexes. Bioinformatics. 2016 10.1093/bioinformatics/btw514 27503228

[40] Berman HM, Westbrook J, Feng Z, Gilliland G, Bhat TN, Weissig H, et al The Protein Data Bank (www.rcsb.org). Nucleic Acids Research. 2000 10.1093/nar/28.1.235 10592235 PMC102472

[41] Waterhouse A, Bertoni M, Bienert S, Studer G, Tauriello G, Gumienny R, et al UCSF Chimera—a visualization system for exploratory research and analysis. The Journal of biological chemistry. 2009 10.1097/MLR.0000000000000625 27623005 PMC5975355

[42] DeLano WL. The PyMOL Molecular Graphics System, Version 1.1. Schr{ö}dinger LLC. 2002. 10.1038/hr.2014.17

[43] Emsley P, Lohkamp B, Scott WG, Cowtan K. Features and development of Coot. Acta Crystallographica Section D: Biological Crystallography. 2010 10.1107/S0907444910007493 20383002 PMC2852313

[44] Yan R, Zhang Y, Li Y, Xia L, Guo Y, Zhou Q. Structural basis for the recognition of SARS-CoV-2 by full-length human ACE2. Science. 2020 10.1126/science.abb2762 32132184 PMC7164635

[45] Holm L, Laakso LM. Dali server update. Nucleic acids research. 2016 10.1093/nar/gkw357 27131377 PMC4987910

[46] Sievers F, Wilm A, Dineen D, Gibson TJ, Karplus K, Li W, et al Fast, scalable generation of high-quality protein multiple sequence alignments using Clustal Omega. Molecular Systems Biology. 2011 10.1038/msb.2011.75 21988835 PMC3261699

[47] van Zundert GCP, Rodrigues JPGLM, Trellet M, Schmitz C, Kastritis PL, Karaca E, et al The HADDOCK2.2 Web Server: User-Friendly Integrative Modeling of Biomolecular Complexes. Journal of Molecular Biology. 2016 10.1016/j.jmb.2015.09.014 26410586

[48] de Vries SJ, Bonvin AMJJ. Cport: A consensus interface predictor and its performance in prediction-driven docking with HADDOCK. PLoS ONE. 2011 10.1371/journal.pone.0017695 21464987 PMC3064578

[49] Sanchez-Garcia R, Sorzano COS, Carazo JM, Segura J. BIPSPI: A method for the prediction of partner-specific protein-protein interfaces. Bioinformatics. 2019 10.1093/bioinformatics/bty647 30020406 PMC6361243

[50] Kozakov D, Hall DR, Xia B, Porter KA, Padhorny D, Yueh C, et al The ClusPro web server for protein-protein docking. Nature Protocols. 2017 10.1038/nprot.2016.169 28079879 PMC5540229

[51] Jiménez-García B, Pons C, Fernández-Recio J. pyDockWEB: A web server for rigid-body protein-protein docking using electrostatics and desolvation scoring. Bioinformatics. 2013 10.1093/bioinformatics/btt262 23661696

[52] Pierce BG, Wiehe K, Hwang H, Kim BH, Vreven T, Weng Z. ZDOCK server: Interactive docking prediction of protein-protein complexes and symmetric multimers. Bioinformatics. 2014 10.1093/bioinformatics/btu097 24532726 PMC4058926

[53] Abraham MJ, Murtola T, Schulz R, Páll S, Smith JC, Hess B, et al Gromacs: High performance molecular simulations through multi-level parallelism from laptops to supercomputers. SoftwareX. 2015 10.1016/j.softx.2015.06.001

[54] Jo S, Kim T, Iyer VG, Im W. CHARMM-GUI: A web-based graphical user interface for CHARMM. Journal of Computational Chemistry. 2008 10.1002/jcc.20945 18351591

[55] Lee J, Cheng X, Swails JM, Yeom MS, Eastman PK, Lemkul JA, et al CHARMM-GUI Input Generator for NAMD, GROMACS, AMBER, OpenMM, and CHARMM/OpenMM Simulations Using the CHARMM36 Additive Force Field. Journal of Chemical Theory and Computation. 2016;12: 405–413. 10.1021/acs.jctc.5b00935 26631602 PMC4712441

[56] Li W, Zhang C, Sui J, Kuhn JH, Moore MJ, Luo S, et al Receptor and viral determinants of SARS-coronavirus adaptation to human ACE2. EMBO Journal. 2005 10.1038/sj.emboj.7600640 15791205 PMC1142572

[57] Wu K, Li W, Peng G, Li F. Crystal structure of NL63 respiratory coronavirus receptor-binding domain complexed with its human receptor. Proceedings of the National Academy of Sciences of the United States of America. 2009 10.1073/pnas.0908837106 19901337 PMC2785276

[58] Park J, Selvaraj B, McShan AC, Boyken SE, Wei KY, Oberdorfer G, et al De novo design of a homo-trimeric amantadine-binding protein. eLife. 2019 10.7554/eLife.47839 31854299 PMC6922598

[59] Lan J, Ge J, Yu J, Shan S, Zhou H, Fan S, et al Structure of the SARS-CoV-2 spike receptor-binding domain bound to the ACE2 receptor. Nature. 2020 10.1038/s41586-020-2180-5 32225176

[60] Laraia L, McKenzie G, Spring DR, Venkitaraman AR, Huggins DJ. Overcoming Chemical, Biological, and Computational Challenges in the Development of Inhibitors Targeting Protein-Protein Interactions. Chemistry and Biology. 2015 10.1016/j.chembiol.2015.04.019 26091166 PMC4518475

[61] Lu L, Liu Q, Zhu Y, Chan KH, Qin L, Li Y, et al Structure-based discovery of Middle East respiratory syndrome coronavirus fusion inhibitor. Nature Communications. 2014 10.1038/ncomms4067 24473083 PMC7091805

[62] Sainz B, Mossel EC, Gallaher WR, Wimley WC, Peters CJ, Wilson RB, et al Inhibition of severe acute respiratory syndrome-associated coronavirus (SARS-CoV) infectivity by peptides analogous to the viral spike protein. Virus Research. 2006 10.1016/j.virusres.2006.03.001 16616792 PMC2582734

[63] Li Q, Zhao Z, Zhou D, Chen Y, Hong W, Cao L, et al Virucidal activity of a scorpion venom peptide variant mucroporin-M1 against measles, SARS-CoV and influenza H5N1 viruses. Peptides. 2011 10.1016/j.peptides.2011.05.015 21620914 PMC7115635

[64] Ke M, Chen Y, Wu A, Sun Y, Su C, Wu H, et al Short peptides derived from the interaction domain of SARS coronavirus nonstructural protein nsp10 can suppress the 2’-O-methyltransferase activity of nsp10/nsp16 complex. Virus Research. 2012 10.1016/j.virusres.2012.05.017 22659295 PMC7114426

[65] Xia S, Yan L, Xu W, Agrawal AS, Algaissi A, Tseng CTK, et al A pan-coronavirus fusion inhibitor targeting the HR1 domain of human coronavirus spike. Science Advances. 2019 10.1126/sciadv.aav4580 30989115 PMC6457931

[66] Bernardi RC, Melo MCR, Schulten K. Enhanced sampling techniques in molecular dynamics simulations of biological systems. Biochimica et Biophysica Acta—General Subjects. 2015 10.1016/j.bbagen.2014.10.019 25450171 PMC4339458

[67] Robert X, Gouet P. Deciphering key features in protein structures with the new ENDscript server. Nucleic Acids Research. 2014 10.1093/nar/gku316 24753421 PMC4086106

