# Supplementary figures and images for: In-silico design of a potential inhibitor of SARS-CoV-2 S protein
Source: PLoS One. 2020 Oct 1;15(10):e0240004. doi: 10.1371/journal.pone.0240004 (PMC7529220; doi:10.1371/journal.pone.0240004)

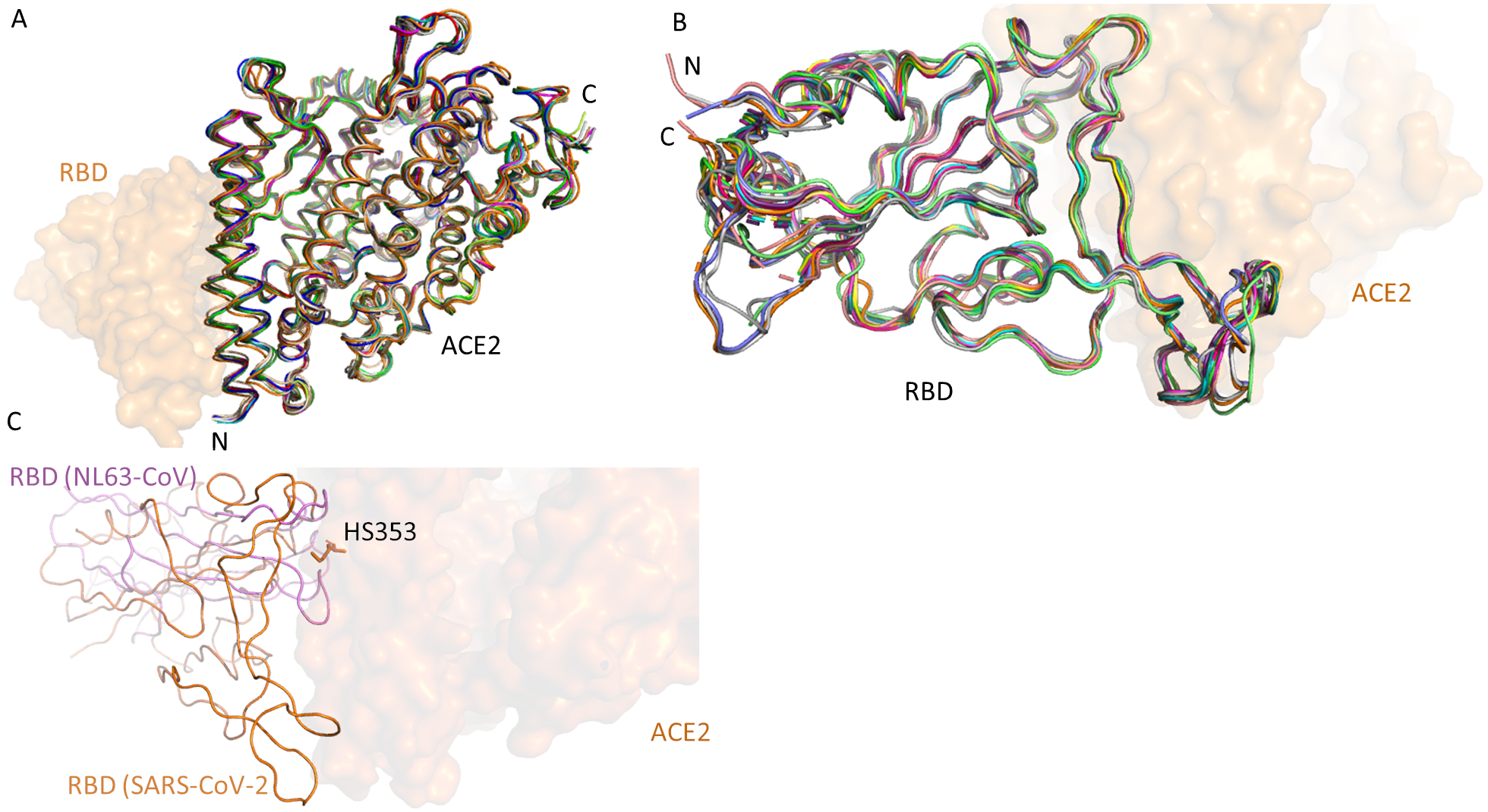

Supplement: S1 Fig — RBD is shown as orange surface. B) alignment of various RBD on pdb 2ajf RBD (grey). Human ACE2 is shown as orange surface. C) NL63 coronavirus (NL63-CoV) RBD structure is unique among coronaviruses. NL63-CoV RBD (3hbk, magenta) binds at hotspot 353 with unique interacting residues. PDB used in A) and B) are: 3d0g (forest), 3kbh (violet), 3sci (limon), 3scj (white), 6cs2 (wheat), 6lzg (red), 6m0j (magenta), 6vw1 (yellow), 6m17 (orange), 3scl (teal), 3d0i (brown), 3d0h (green), 3sck (blue) and 6acg (cyan). (TIF) [file pone.0240004.s001.tif]

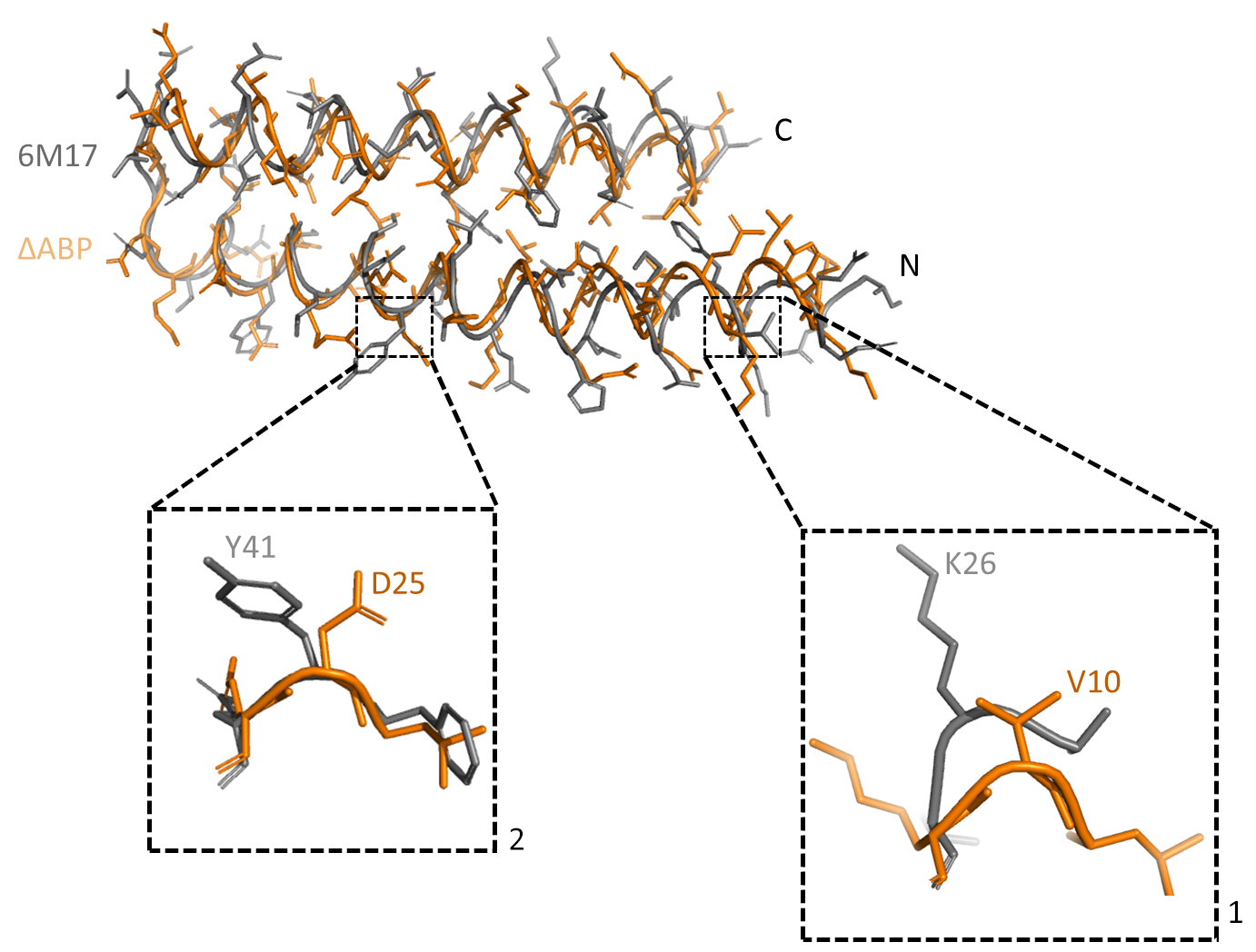

Supplement: S2 Fig — Inset1: Close up view of residue Val10/Lys26. Inset 2: Close up view of Asp25/Tyr41. (TIF) [file pone.0240004.s002.tif]
